# Evaluation of pyrimidine-based compounds as AChE and BChE inhibitors: in vitro inhibition, molecular modeling, and statistical evaluation

**DOI:** 10.1007/s00210-026-05347-0

**Published:** 2026-04-23

**Authors:** Zuhal Alım, Yeliz Demir

**Affiliations:** 1 Department of Chemistry, Faculty of Arts and Sciences, Kırşehir Ahi Evran University, Kırşehir, 40100 Türkiye; 2 Department of Molecular Biology and Genetics, Faculty of Arts and Sciences, University of Kırşehir Ahi Evran, Kırşehir, 40100 Türkiye; 3 Department of Pharmacy Services, Nihat Delibalta Göle Vocational High School, Ardahan University, Ardahan, Türkiye; 4 Faculty of Science, Department of Chemistry, Atatürk University, Erzurum, Türkiye

**Keywords:** Acetylcholinesterase, Butyrylcholinesterase, Pyrimidine, Inhibition, Molecular docking

## Abstract

Alzheimer’s disease (AD) is a neurodegenerative disease characterized by dementia, particularly in older adults. It is a process that is increasing significantly with the aging population worldwide, has yet to be cured, and therefore challenges healthcare systems. The ability of acetylcholinesterase (AChE) and butyrylcholinesterase (BChE) inhibitors to modulate neurotransmitter levels has made AChE/BChE inhibitors central therapeutic targets in drug development studies for the treatment of AD. Previous studies have demonstrated the beneficial effects of pyrimidine derivatives on cognitive functions and highlighted their high therapeutic potential against neurodegenerative diseases. Considering the pharmacological importance of AChE/BChE inhibitors and pyrimidine derivatives, this study investigated the inhibitory potential of seven different pyrimidine derivatives (**1–7**) on AChE and BChE using both in vitro and in silico approaches. Analysis of IC_50_ values indicated that compounds **1–7** (IC_50_: 14.89–77.70 nM) exhibited strong inhibitory effect. Compound **6** (IC_50_:14.89 nM) had the strongest inhibitory effect on AChE, while it showed a much weaker inhibitory effect against BChE (IC_50_: 357 nM), corresponding to an approximately 24-fold selectivity for AChE. Molecular modeling results indicate that compounds **6** and **7** exhibit favorable interactions within the active site of the enzyme. In addition, compounds **1** and **3**, which exhibited the strongest inhibitory effects on BChE, appear to display a multiple binding profile with the active site of BChE. Correlation and regression analyses indicated that compounds **1–7** display a structure–activity relationship (SAR) consistent with strong inhibitory potency toward AChE, while showing comparatively weaker inhibition toward BChE.

**Supplementary Information:**

The online version contains supplementary material available at 10.1007/s00210-026-05347-0.

## Introduction

Alzheimer’s disease (AD) is a progressive neurodegenerative disease, the most common form of dementia, and with the aging world population, it is increasingly placing a burden on the global public health system (Kumar et al. 2015; Kamatham et al. 2024; Fayed et al. 2025). The World Alzheimer’s Report, published in 2022, reported that there are more than 55 million people with Alzheimer’s disease worldwide, and that this number is expected to reach 82 million by 2030 and 138 million by 2050 (Khedraoui et al. 2025). Because AD is such a complex disease, decades of research have not yet fully elucidated its pathogenesis (Kim et al. 2022). The main neuropathological hallmarks of AD are the accumulation of extracellular β-amyloid (Aβ) plaques and intracellular neurofibrillary tangles (NFTs) composed of hyperphosphorylated tau protein. While the amyloid cascade hypothesis remains the dominant approach in the field, studies aimed at elucidating the pathogenesis of AD suggest that various neurotransmitter systems and cellular pathways also play a role in the disease, highlighting the need for multi-targeted treatment strategies (Kamatham et al. 2024; Khedraoui et al. 2025).

Degeneration of cholinergic neurons in the basal forebrain is one of the earliest and most prominent abnormalities observed in AD. This leads to reduced acetylcholine (ACh) levels, particularly in brain regions associated with memory and cognitive functions (Chen et al. 2022; Demir et al. 2025). Acetylcholinesterase (AChE; EC 3.1.1.7) and butyrylcholinesterase (BChE; EC 3.1.1.8) are two important cholinesterase enzymes that regulate ACh levels in the synaptic cleft. AChE rapidly terminates cholinergic signaling by hydrolyzing ACh to choline and acetic acid (Jovičić 2024, Tokalı et al. 2025, 2026). BChE, which has a broader tissue distribution than AChE, shares structural and functional similarities with AChE, but its physiological function has not yet been elucidated (Silman 2021; Hajimohammadi et al. 2025).

Notably, as AD progresses, AChE activity decreases by up to 45% in certain brain regions, whereas BChE activity increases by up to 90%. These opposing changes in enzymatic activity suggest that BChE may play a compensatory role in regulating ACh levels during the later stages of the disease, making it a potential therapeutic target (Grossberg 2003; Yu et al. 2024; Sharma et al. 2024; Khedraoui et al. 2025). Currently approved AChE inhibitors provide only temporary and limited cognitive improvements and do not halt disease progression. At the same time, the limited efficacy and serious side effects of these inhibitors approved for AD treatment necessitate the need for new inhibitor candidates that are more selective, have fewer side effects, and are highly effective (Ruangritchankul et al. 2021; Hossain et al. 2025). Consequently, the development of dual-action inhibitors targeting both AChE and BChE has emerged in recent years as a promising therapeutic strategy (Elsawalhy et al. 2024; Cavallaro et al. 2025). These agents aim to enhance cholinergic neurotransmission more comprehensively while reducing peripheral toxicity, particularly through BChE selectivity.

Pyrimidine is an organic compound with a six-membered aromatic and heterocyclic ring structure, containing nitrogen atoms at positions 1 and 3. This structure, consisting of four carbon atoms and two nitrogen atoms in alternating positions, is of significant importance in drug design due to its structural and electronic properties (Mosaad et al. 2022; Nammalwar and Bunce 2024; Marzouk 2025). The pyrimidine skeleton is also found in the structures of fundamental biomolecules such as nucleic acid bases (cytosine, thymine, uracil), certain vitamins (thiamine, folic acid), and alkaloids, and is considered a privileged pharmacophore structure in medicinal chemistry due to its biocompatibility and pharmacological diversity (Mishra and Tomar 2011; Sharma et al. 2014; Venugopala and Kamat 2024).

In modern drug design, pyrimidine derivatives have emerged as prominent frameworks in drug development due to their unique physicochemical properties and broad biological activity profiles (Venugopala and Kamat 2024; Marzouk 2025). Their electron-deficient ring systems allow for diverse non-covalent interactions with biological targets, their metabolic stability prevents rapid degradation in vivo, and strategic substitutions at the C2, C4, and C6 positions enable fine-tuning of target selectivity, making them highly valuable from a pharmaceutical perspective (Taylor et al. 2014; Xiao et al. 2023; Alsharif et al. 2023; Marzouk 2025).

Pyrimidine derivatives exhibit a wide range of pharmacological activities, including antiproliferative (Cortes-Percino et al. 2019; Eldeeb et al. 2025), antiviral (Farghaly et al. 2023), anticancer (Tylinska et al. 2021), anti-inflammatory (Keshk et al. 2025), antibacterial (Ahmed et al. 2023), antifungal (Coelho et al. 2024), antiglucuronidase (Barakat et al. 2016), antidiabetic (Marzouk 2025) and antitubercular effects (Finger et al. 2023). Furthermore, Piribedil, a molecule with a pyrimidine skeleton, is used in the treatment of Parkinson’s disease. Piribedil has become a prominent example highlighting the neuroprotective potential of pyrimidine-based molecules (Pant et al. 2022; Balli et al. 2025). Additionally, numerous studies in the literature highlight the potential of various pyrimidine derivatives to exhibit anti-Alzheimer activity through inhibition of AChE and BChE enzymes (Mohamed et al. 2011; Ahmad et al. 2016; Jain et al. 2021; Bortolami et al. 2021; Han et al. 2022).

**Fig. 1:**
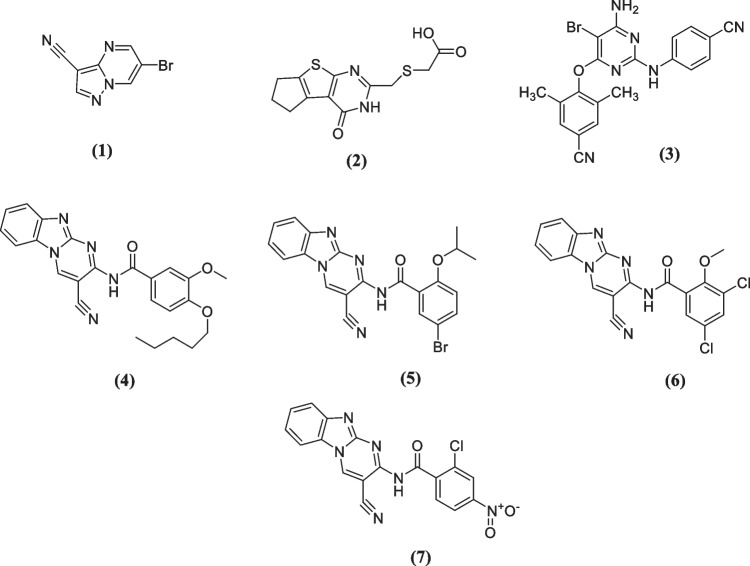
The chemical structures of the pyrimidine molecules used in this study

Given the therapeutic potential of pyrimidine derivatives and the pharmacological importance of AChE/BChE inhibitors, this study investigated the inhibitory potential of some pyrimidine derivatives (**1–7**) (Fig. 1) on AChE/BChE through in vitroand in silico approaches. The compounds used in the present study were not synthesized from a precursor compound, but rather selected from an existing pyrimidine-based structural framework to represent targeted substitution diversity. The reason for investigating these pyrimidine derivative molecules as AChE/BChE inhibitor candidates in this study is that these compounds possess heteroaromatic, planar, electron-rich, hydrogen-bonding, and aromatic interaction structural characteristics suitable for interacting with the active and peripheral anionic sites of cholinesterase enzymes. The results suggest that these molecules can contribute to studies on the synthesis of pyrimidine derivative cholinesterase inhibitors.

## Materials and methods

### Chemicals

Pyrimidine derivatives ((**1:** 6-Bromopyrazolo[1,5-a] pyrimidine-3-carbonitrile (CAS No: 352637–44-6), **2:** ([(4-Oxo-3, 5,6,7-tetrahydro-4H-cyclopenta[4,5]thieno[2,3-d]pyrimidin-2-yl)methyl]thio)acetic acid (Product No: CDS012972), **3:** 4-((6-Amino-5-bromo-2-((4-cyanophenyl)amino)pyrimidin-4-yl)oxy)−3,5-dimethylbenzonitrile (CAS No: 269055–15-4), **4:** N-(3-cyano -benzo (4,5) imidazo (1,2-a) pyrimidin-2-yl)−3-meo-4-pentyloxy-benzamide (CAS No: 116477–75-9), **5:** 5-Br-N-(3-cyano-benzo (4,5)) imidazo (1,2-a) pyrimidin-2-yl)−2-isopropoxy-benzamide (CAS No: 116477–62-4), **6:** 3,5-dichloro-N-(3-cyano-benzo (4,5) imidazo (1,2-a)pyrimidin-2-yl)−2-meo-benzamide (CAS No: 116477–82-8), **7:** 2-chloro-N-(3-cyano-benzo (4,5) imidazo (1,2-a)pyrimidin-2-yl)−4- nitro-benzamide) (CAS No: 116477–89-5)) and all chemicals used in purification processes and kinetic studies were purchased from Sigma-Aldrich Co. (Steinheim, Germany). In addition, AChE from *Electrophorus electricus* (electric eel) and BChE from equine serum (AChE: CAS no. 9000–81-1; BChE: CAS no. 9001–08-5) were purchased from Sigma-Aldrich Co.

#### In vitro inhibition studies

The inhibitory effects of test compounds against AChE and BChE were determined spectrophotometrically using the modified Ellman method (1961) as commonly applied in cholinesterase inhibition studies (Koksal et al. 2019; Tunc et al. 2025). The reaction mixtures were prepared in 50 mM Tris–HCl buffer (pH 8.0) at room temperature (25 °C). AChE (*Electric eel*) and BChE (equine serum) enzymes were used, and the reactions were initiated by addition of respective substrates, acetylthiocholine iodide and butyrylthiocholine iodide. The final concentrations of substrates and DTNB (5,5′-dithiobis-(2-nitrobenzoic acid)) were 0.5 mM and 0.3 mM, respectively, and enzyme activity per well was adjusted to achieve a linear reaction rate. Test compounds were dissolved in DMSO, with the final solvent concentration not exceeding 1% (v/v) to avoid enzyme inhibition by solvent. After incubation with enzyme for 10 min, absorbance was recorded at 412 nm. IC_50_ values were calculated using nonlinear regression and results are reported as mean ± standard deviation of at least three independent determinations.

#### Multivariate correlation, regression and selectivity assessment of cholinesterase inhibitory profiles

All statistical analyses and graphical outputs were performed in R software (version 4.5.1) to ensure full reproducibility. Raw IC_50_ data for AChE and BChE were imported using read.csv() or readxl::read_excel(). Prior to analysis, data integrity was verified through inspection of value ranges, consistency of compound labeling, and identification of missing or duplicated entries.

Descriptive statistics were obtained using standard summary measures, and data distributions were visually screened using boxplots to assess dispersion and potential extreme observations. Given the small sample size, normality tests were applied cautiously and used for descriptive purposes only. Bivariate associations between inhibition metrics were assessed using Pearson correlation coefficients, with the Spearman rank correlations additionally reported where distributional assumptions were not met. To examine agreement between assay-derived measures, both ordinary least squares (OLS) regression and the Deming regression were employed. OLS models were fitted using the lm() function, while the Deming regression accounting for measurement error in both variables was performed using the mcr::mcreg() or MethComp::Deming() functions with equal error variance assumed. Selectivity indices were calculated directly in R as ratios of IC_50_(BChE)/IC_50_(AChE) and integrated with correlation analyses to distinguish AChE-selective versus dual-inhibitory profiles. All figures were generated using ggplot2.

### Molecular docking

Molecular docking analyses were performed utilizing AutoDock Vina software (Trott and Olson 2010). The crystal structures of *Tetronarce californica* AChE (PDB ID: 1EVE) and Homo sapiens BChE (PDB ID: 5NN0) were sourced from the RCSB Protein Data Bank (https://www.rcsb.org). Although in vitro inhibition assays were conducted using *Electrophorus electricus* AChE, molecular docking was performed using the AChE structure from *Tetronarce californica* (PDB ID: 1EVE). This approach is well established in the literature, as cholinesterases from electric eel, torpedo ray, and human sources exhibit a high degree of sequence and structural conservation, particularly within the catalytic triad, peripheral anionic site, and the active-site gorge that governs ligand recognition and binding. High-resolution co-crystal structures of *E. electricus* AChE are not available, whereas TcAChE provides structurally reliable and extensively validated templates for mechanistic docking studies. Accordingly, TcAChE has been widely used as a surrogate model to rationalize inhibition data obtained from electric eel AChE assays, with docking results interpreted as qualitative and mechanistic rather than species-specific quantitative predictions (Sussman et al. 1991; Silman et al. 2005; Colović et al. 2013).

A grid box measuring 40 × 40 × 40 points, with a spacing of 0.375 Å, was centered on the anticipated binding sites of each target. For AChE (PDB ID: 1EVE), the grid box center was defined at *x* = 16.162, *y* = 39.021, *z* = 41.281, covering the entire catalytic gorge including both the catalytic anionic site (CAS) and the peripheral anionic site (PAS). For BChE (PDB ID: 5NN0), the grid box center was set at *x* = 2.132, *y* = 56.598, *z* = 94.400, ensuring full coverage of the active-site cavity. The docking poses exhibiting the most advantageous binding energies (minimum binding free energy) were chosen for comprehensive interaction investigation. In the target preparation phase utilizing AutoDock Tools, polar hydrogen atoms were included, and Kollman charges were allocated to the protein targets. Ligands were generated by applying Gasteiger charges and delineating rotatable bonds. The interactions between the ligands and the crystal structures of AChE and BChE were visualized and evaluated with Discovery Studio Visualizer (Version 4.1, Accelrys Software Inc., San Diego, CA, USA). To validate the reliability of the docking protocol, a re-docking procedure was performed using the co-crystallized ligands present in the crystal structures of the target enzymes. The native ligands were first removed from the binding sites of TcAChE (PDB ID: 1EVE) and BChE (PDB ID: 5NN0), and subsequently re-docked using the same grid parameters and docking settings employed for the tested pyrimidine derivatives. The predicted ligand poses were then compared with the corresponding crystallographic conformations by calculating the root mean square deviation (RMSD) values.

## Result and discussion

### AChE and BChE enzymes inhibition and structure–activity relationship (SAR) analysis

Investigation of the pyrimidine-derived inhibition of AChE and BChE showed an unequivocal correlation between structure and activity. Small changes in the peripheries of the molecules produced different trends on how they inhibited these enzymes. Tacrine (THA) is still a standard reference owing to it being tightly bound by BChE (IC_50_: 5.22 nM) (Table 1), halogenation, methoxylation, and alkoxylation of our compounds maximally changed their mode of action with cholinesterase through different mechanisms involving size and electronic properties.

**Table 1:** Inhibition results of compounds **1–7** on AChE and BChE

Compounds	IC_50_ for AChE (nM)	IC_50_ for BChE (nM)
1	43.75 ± 6.29	175.00 ± 4.11
2	25.00 ± 0.29	441.30 ± 35.52
3	77.70 ± 1.48	175.00 ± 32.28
4	26.92 ± 2.69	460.80 ± 48.61
5	24.14 ± 1.22	273.97 ± 10.34
6	14.89 ± 2.00	357.00 ± 48.61
7	20.58 ± 2.17	344.83 ± 48.89
Tacrine (THA)*	25.00 ± 1.24	5.22 ± 0.22

^*^Tacrine (THA) used as reference inhibitor for AChE and BChE

Compound **1** showed inhibitory values of IC_50_:43.75 nM for AChE and IC_50_:175 nM for BChE (Figure S1). The presence of the bromine substituent was associated with moderate AChE inhibitory potency (IC_50_: 43.75 nM). Docking analysis revealed π–halogen and hydrophobic contacts with aromatic residues, providing a structural rationale for the observed inhibitory effect. Although compound **1** exhibited moderate inhibitory effect compared with the reference inhibitor THA (25 nM), the docking results suggest that the molecule primarily interacts within the CAS and shows limited interaction with residues associated with the PAS. This interaction pattern may partially explain the relatively lower inhibitory potency observed for this compound.

Compound** 2** showed a much stronger inhibition of AChE at IC_50_:25 nM (Figure S1, Table 1), which is similar to THA, but a much weaker inhibitory potency for BChE at 441.30 nM (Table 1). The addition of a thioacetic group was thought to be responsible for this improvement. Hydrophobic interactions within the CAS region and potential hydrogen-bonding contacts near the peripheral sitemay help rationalize the observed inhibitory potency.

Compound **3** showed IC_50_ values of 77.70 nM for AChE and 175 nM for BChE (Table 1, Figure S2). The conformational flexibility of the molecule in AChE and BChE binding, despite its steric complexity, allows it to adapt to the target site and contribute to the observed inhibitory potency. While bromine and cyano groups are generally expected to provide pairwise interactions via π-electron attraction and potential hydrogen bonding, increased steric complexity prevented optimal alignment within the gorge (the deep groove in the enzyme’s active site), thus reducing efficacy. This observation suggests that structurally complex yet conformationally adaptable molecules may retain effective inhibitory potency toward cholinesterase enzymes. Compound** 3**, also known as Etravirine, is an US FDA-approved HIV-1 non-nucleoside reverse transcriptase inhibitor. Etravirine binds directly to reverse transcriptase and inhibits its activity by disrupting the enzyme's catalytic site. Thanks to its flexibility in the binding site, it can exhibit high activity without being affected by mutations. Crystallography and molecular modeling studies show that the molecule adapts by stretching, repositioning, and orienting itself within its binding site (Haubrich et al. 2008; Deeks and Keating 2008). These results suggest that structurally complex but flexible molecules like etravirine can be effective inhibitors at different enzyme targets and offer the advantage of rapid binding. The IC_50_ value for compound **4** was determined to be 26.92 nM for AChE and 460.80 nM for BChE (Table 1), indicating that this compound predominantly behaves as an AChE-selective inhibitor.

Compound **5** has exhibited AChE inhibition of IC_50_:24.14 nM (Figure S1, Table 1) and BChE inhibition of IC_50_:273.97 nM (Table 1). This is indicative of an optimal balance between steric encumbering and productive accommodation in the spatial configuration of the molecule. Replacing the methoxy group with an isopropoxy moiety results in a more stable hydrophobic insertion into the catalytic gorge of the enzymes which allows for deeper π–π interaction and protection of beneficial interactions from solvent exposure. This structural enhancement is related to activities that exceed THA against AChE.

Compound **6** demonstrated the best AChE inhibition at IC_50_: 14.89 nM (Figure S1, Table 1), while the BChE value was 357 nM (Table 1). Compound **7** exhibited IC_50_ values of 20.58 nM for AChE (Figure S1, Table 1) and 344.83 nM for BChE (Table 1), was observed to fall within a narrow spectrum between advantageous polarity and excessive electronic density. The nitro substituent was powerful as it was electron withdrawing, and polar bonding was possible; however, it was positioned ortho to the chlorine and therefore intramolecular strain was present.

Compounds **1** and **4–7** can be considered within a related structural framework, as they share a pyrimidine-based aromatic core while differing in the nature and position of their peripheral substituents. Within this subgroup, compound **1**, bearing a relatively simpler bromopyrazolo-pyrimidine scaffold, showed only moderate AChE inhibitory potency, whereas compounds **4–7**, which contain more extended fused aromatic/benzamide-type architectures, generally exhibited stronger AChE inhibition. This trend suggests that increasing scaffold extension and peripheral hydrophobic functionality may improve complementarity with the elongated AChE catalytic gorge. In particular, methoxy, isopropoxy, dichloro, and nitro-containing substituents in compounds **4–7** may enhance hydrophobic packing, aromatic stacking, and positional stabilization within the gorge, thereby favoring stronger AChE inhibitory potency. In contrast, despite the broader active-site cavity of BChE, these same structural features did not translate into comparable BChE inhibition, suggesting that the substituent pattern in this series is better suited to the topological and interaction requirements of AChE than BChE.

**Fig. 2:**
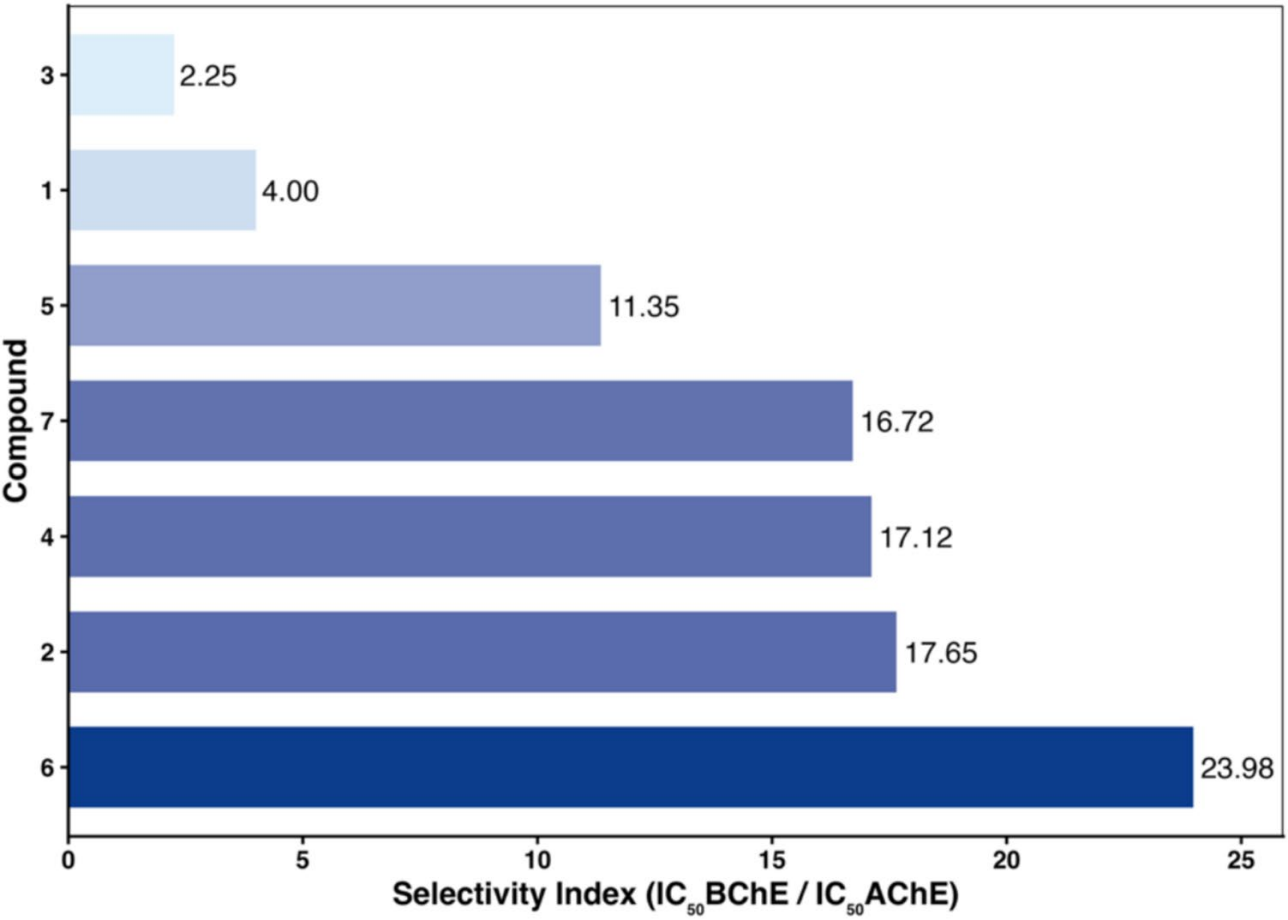
Selectivity index (IC_50_(BChE)/IC_50_(AChE)) of the tested compounds. Higher values indicate increased selectivity toward AChE inhibition, whereas lower values reflect relatively stronger BChE inhibition

The selectivity index, calculated as the ratio IC_50_(BChE)/IC_50_(AChE) using raw IC_50_ values, revealed clear differences in enzyme preference among the tested compounds. Higher selectivity index values indicate stronger preference toward AChE inhibition, whereas lower values suggest relatively enhanced BChE inhibition. Based on this definition, compound **6** exhibited the highest AChE selectivity, while compound **3** showed the lowest selectivity, reflecting a more balanced inhibitory profile (Fig. 2). These findings may assist in the rational design of subtype-selective cholinesterase inhibitors with reduced off-target effects and improved safety profiles.

#### Correlation, regression, and selectivity analysis of cholinesterase inhibition

### Exploratory correlation and regression analysis

**Fig. 3:**
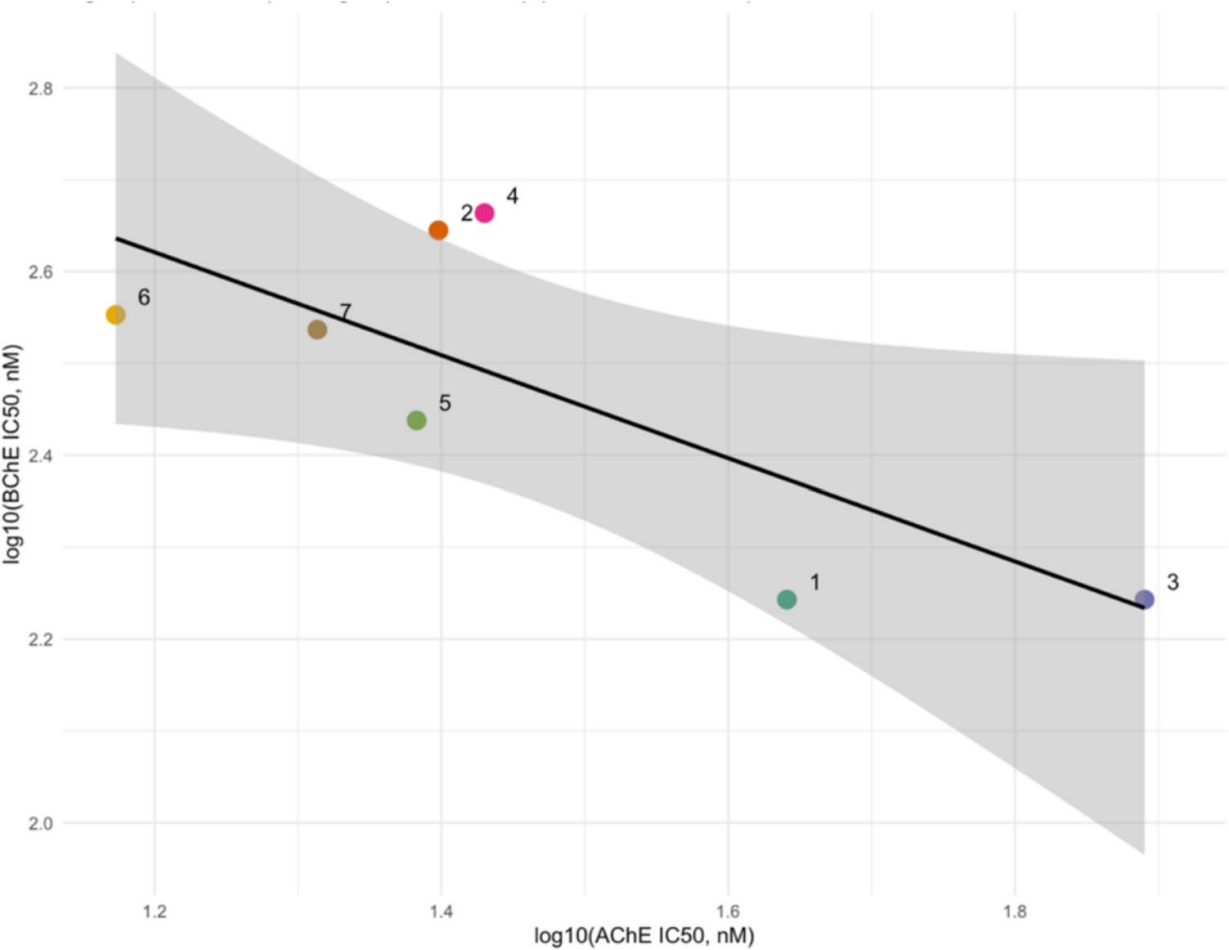
Correlation of AChE and BChE inhibition potency of the compounds The IC_50_values were transformed to log10 scale for both enzymes and a scatter plot was constructed where each point represents an individual compound

To explore the relationship between AChE and BChE inhibitory potencies across the tested compounds, bivariate correlation and regression analyses were performed using the IC_50_ values reported in Table 1. Given the limited number of compounds (*n* = 7), all statistical results were interpreted within an exploratory framework. The Pearson and Spearman correlation coefficients were first calculated using raw IC_50_ values. On the raw scale, Pearson analysis indicated a moderate inverse association between AChE and BChE inhibition (*r* = –0.697), although this relationship did not reach statistical significance (*p* = 0.081; Table 2). Similarly, Spearman’s rank correlation suggested a weak-to-moderate monotonic inverse trend (*ρ* = –0.414, *p* = 0.355), indicating that compounds exhibiting stronger AChE inhibition tended to show weaker BChE inhibition, albeit with substantial variability. To reduce scale effects and stabilize variance, the Pearson correlation was additionally evaluated using log₁₀-transformed IC_50_ values. Under this transformation, the inverse association became more pronounced (*r* = –0.755) and reached borderline statistical significance (*p* = 0.049; Table 2, Fig. 3). This result should be interpreted cautiously, as the transformation affects variance structure and the sample size remains limited.

**Table 2:** Comparison of the Pearson and Spearman correlations on raw vs log_10_ (IC_50_) data

Analysis scale	Correlation type	* r/ρ* value	*p*-value	Interpretation
Raw IC_50_	Pearson *r*	–0.697	0.081	Not significant
Raw IC_50_	Spearman *ρ*	–0.414	0.355	Not significant
log_10_(IC_50_)	Pearson *r*	–0.755	0.049*	Borderline significant
log_10_(IC_50_)	Spearman *ρ*	–0.414	0.355	Not significant

### Deming and OLS correlation model for AChE and BChE (log_10_ IC_50_)

**Fig. 4:**
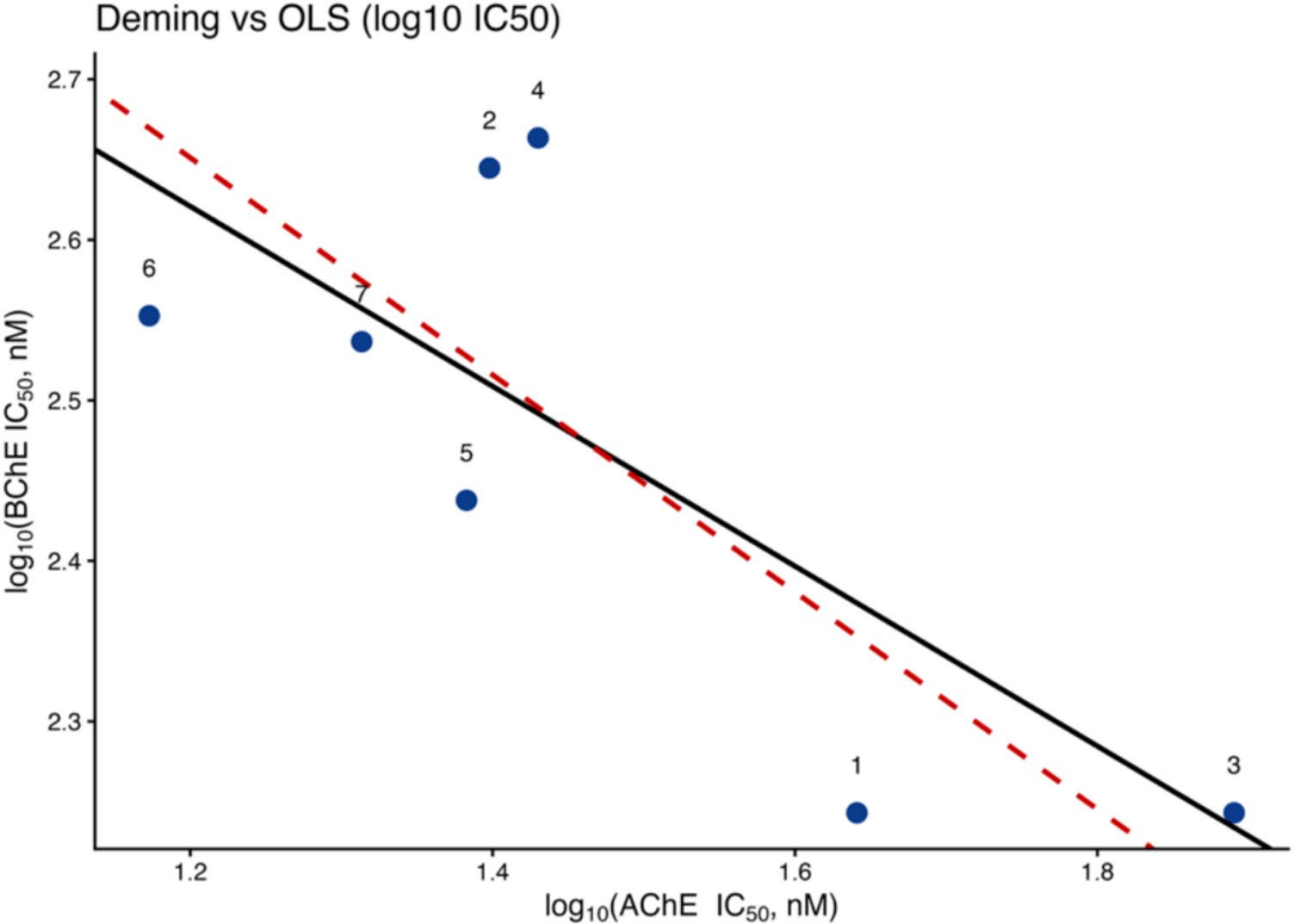
Results OLS versus Deming regression Analysis of log_10_-transformed IC_50_ values obtained from AChE and BChE inhibition. Each blue dot represents a unique compound and the solid black line (OLS) assumes error-free AChE measurements. The dashed red (Deming) line includes measurement error in both enzymes

In order to further illustrate the direction and magnitude of this association, OLS and Deming regression analyses were applied to log_10_-transformed IC_50_ values (Fig. 4). OLS regression yielded a negative slope (*β* ≈ –0.56; *R*^2^≈ 0.57), suggesting that increased potency toward AChE tended to coincide with reduced potency toward BChE. Deming regression, which accounts for measurement error in both variables, produced a comparable negative slope (*β* ≈ –0.68). In the absence of independent estimates of assay variance, the error variance ratio (λ) was set to 1, assuming similar experimental uncertainty for AChE and BChE measurements. Taken together, the correlation and regression analyses consistently point toward a tendency for inverse selectivity between AChE and BChE inhibition across the tested compounds. However, given the small sample size and borderline *p*-values, these findings should be regarded as indicative rather than confirmatory and primarily serve to support qualitative structure–activity interpretation.

### Molecular docking

#### Docking interaction analysis for AChE

**Fig. 5:**
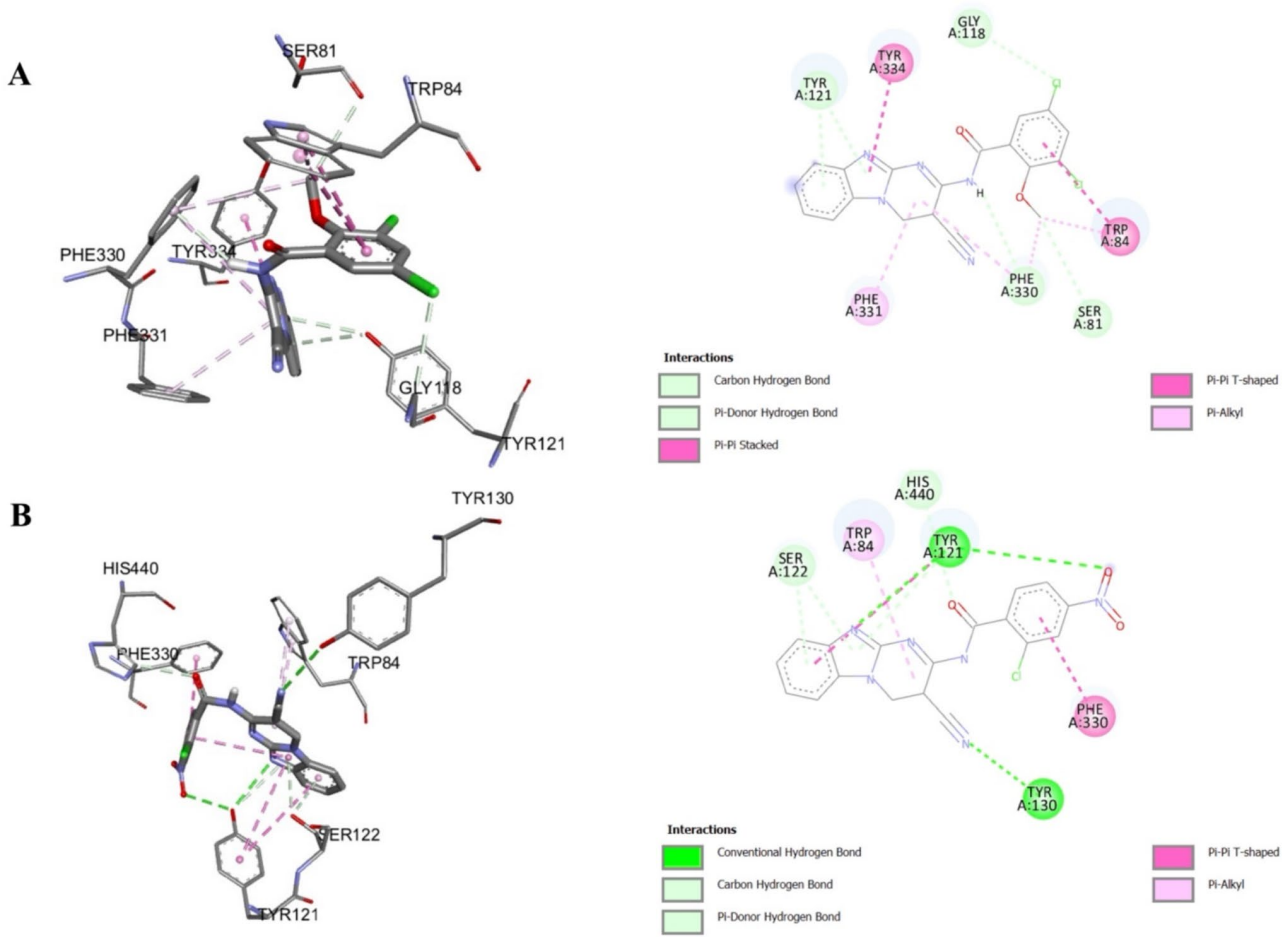
Three-dimensional (3D) and two-dimensional (2D) pose **A** Compound **6** and **B** Compound **7**

The docking energy of compound **6** was predicted to be –10.5 kcal/mol and exhibited a much stronger binding to AChE, which accounted for the stable and favorable ligand–enzyme complex. The ligand is deeply buried within the catalytic gorge and binds to both the CAS and PAS; a binding pattern usually associated with potent inhibitory effect toward cholinesterase enzymes. The aromatic headpiece of the ligand docks at the active site, causing a notable π–π stacking with Trp84 which is a substrate recognition residue. Additional π –π interactions with Tyr334 further augmented arene stabilization, whereas π –alkyl contacts with Phe331 assisted in preserving hydrophobic packing for ligand anchoring (Fig. 5A, Table 3). Notably, hydrogen bond interactions were observed with Ser81, Phe330, Gly118, and Tyr121 which would imply potential interference with the catalytic cycle. This hydrogen bond is important and may impede the hydrolysis of AChE by preventing nucleophilic activation in the esteratic subsite. (Fig. 5A, Table 3). This dual mode of binding is pharmacologically important due to the capability of potentially inhibiting acetylcholine hydrolysis while also concomitantly blocking AChE-mediated Aβ aggregation which may be advantageous for a dual therapy in AD.

**Table 3:** Key interactions of compounds **6** and **7** with AChE identified by molecular docking

Compound	Residue	Interaction type	Binding region
6	Trp84	π-π T-shaped	CAS
6	Tyr334	π-π stacking	CAS
6	Phe330	Hydrogen bond	CAS
6	Tyr121	Hydrogen bond	Peripheral gorge
6	Ser81	Hydrogen bond	Catalytic gorge
7	Tyr121	Conventional hydrogen bond	Peripheral gorge
7	Tyr130	Conventional hydrogen bond	Peripheral gorge
7	Trp84	π–alkyl	CAS
7	Phe330	π-π stacking	CAS
7	Ser122	Hydrogen bond	Catalytic gorge

Compound **7** was predicted to have a docking score of –11.3 kcal/mol and demonstrated an even more stabilized interaction within the AChE active-site gorge compared to other representatives of the series. Unlike the compound **6**, this compound engages in a richer interaction network comprising hydrogen bonding, π–cation, and π–π T-shaped contacts, suggesting a strongly anchored inhibitory profile. A classical hydrogen bond was established with Tyr121 and Tyr130 located in the PAS that may be implicated also in the inhibition of AChE-induced Aβ aggregation. Furthermore, it is stabilized by a π–donor hydrogen bond with Ser122 at the entry of the catalytic gorge. These contacts hold the ligand in a planar conformation suitable for dual binding sites (Fig. 5B, Table 3). At the mid-gorge, it is stabilized through edge-to-face π–π stacking interaction with Phe330. In addition, π–alkyl interactions with Trp84 increase the hydrophobic snugness which may account for low binding energy (Fig. 5B, Table 3). To ensure the accuracy of docking study, validation was carried by re-docking the co-crystallized ligand (donepezil) into TcAChE active site. The predicted binding energy (ΔG) of the redocked donepezil was − 11.3 kcal/mol.

**Fig. 6:**
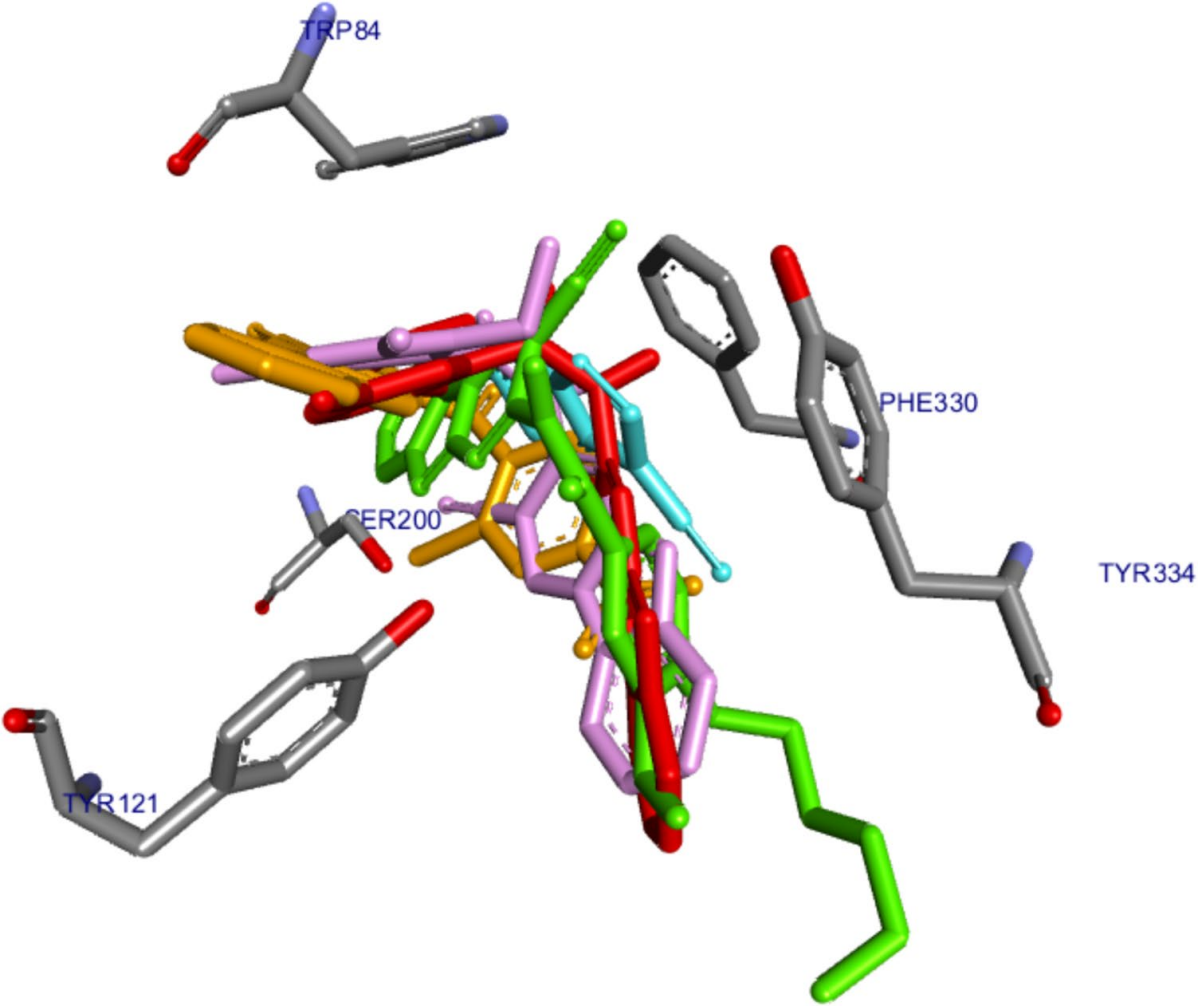
Superimposed docking poses of compounds **1** (blue), **4** (green), **5** (red), **6** (purple) and **7** (orange) within the catalytic gorge of AChE (PDB ID: 1EVE). The common scaffold of the compounds occupies a conserved region of the active-site gorge, while differences in substituent orientation lead to distinct interactions with surrounding residues such as Trp84, Tyr121, Ser200, Phe330, Tyr334

To further explore the SAR among the most active compounds, the docking poses of compounds **1**,** 4**,** 5**,** 6**, and **7** were superimposed within the catalytic gorge of AChE (Fig. 6). The overlay analysis revealed that the shared core scaffold of these molecules occupies a conserved region within the enzyme active site. However, differences in the orientation of the substituent groups result in distinct interactions with surrounding residues lining the catalytic pocket, which may contribute to the observed differences in inhibitory effect. These findings suggest that the conserved positioning of the common scaffold enables stable accommodation within the catalytic gorge, while substituent-dependent interactions may modulate binding strength and inhibitory potency.

### Docking interaction analysis for BChE

**Fig. 7:**
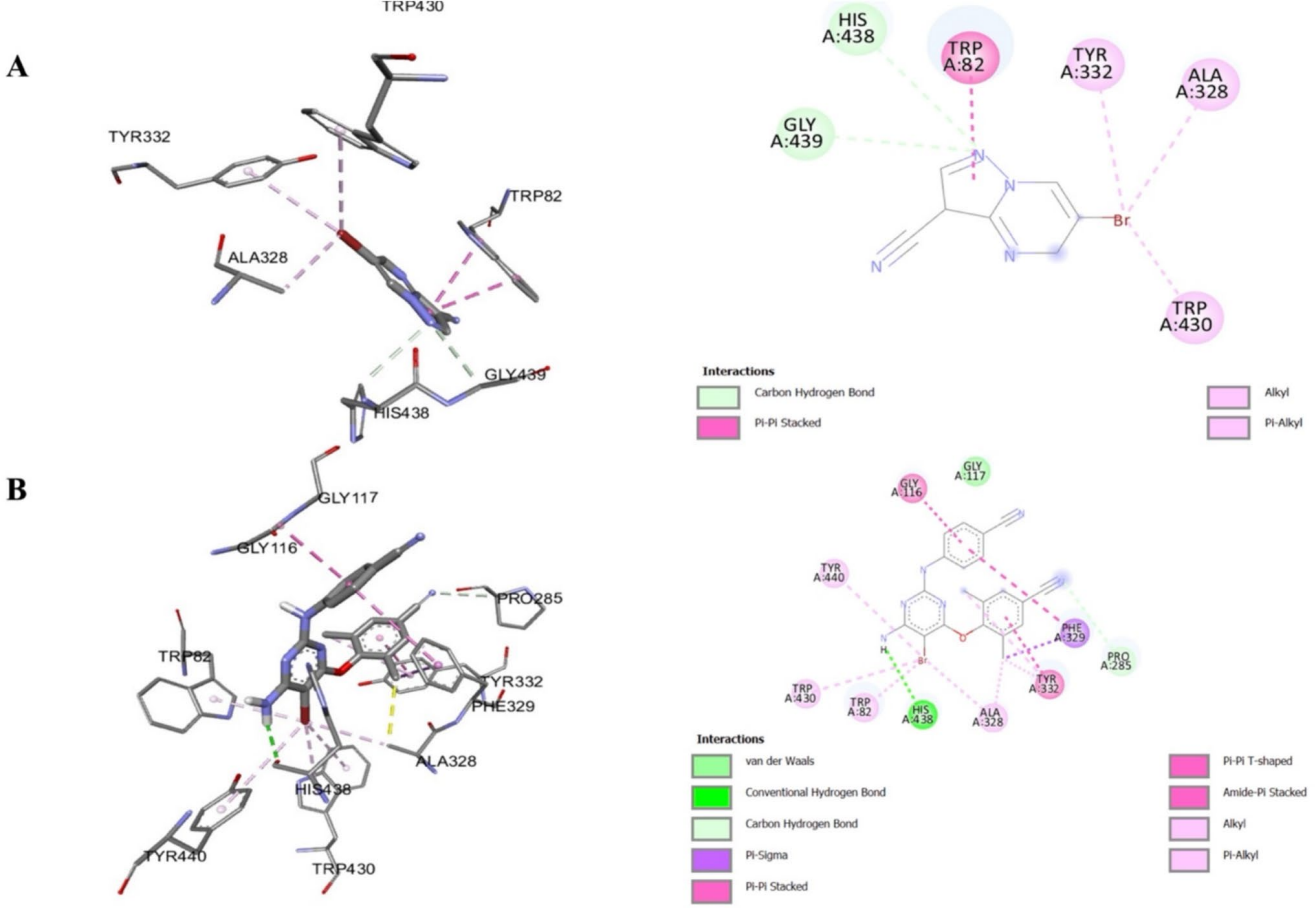
Three-dimensional (3D) and two-dimensional (2D) pose **A** Compound **1** and **B** Compound **3** for BChE

Compound **1** showed relatively lower interaction pattern in the BChE active gorge region (–6.4 kcal/mol). Compared with the high-inhibitory potency analogues (ΔG < –10 kcal/mol), this compound almost interacted solely by π–π stacking to Trp82 and by few π–alkyl contacts to Ala328, Tyr332 and Trp430 as well as single CH bond. Hydrogen bond contacts to the essential catalytic/peripheral residues (including His438 and Gly 439) indicates inadequate binding energy in the enzyme’s double sub-sites (Fig. 7A, Table 4). Moreover, the ligand is also situated in a more superficial position in the gorge entrance (as opposed to a deeper channel into the catalytic region) suggesting an incorrect orientation and less complementarity with respect to hydrophobic pocket. The mode of interaction is van der Waals driven, devoid of directional bond network for strong inhibitory potency.

**Table 4:** Key interactions of compounds **1** and **3** with BChE identified by molecular docking

Compound	Residue	Interaction type	Binding region
1	Trp82	π-π stacking	CAS
1	Trp430	π- alkyl	Active-site gorge
1	Tyr332	π-π stacking	CAS
1	Ala328	Alkyl	Active-site gorge
1	His438	Carbon Hydrogen bond	Catalytic site
1	Gly439	Carbon Hydrogen bond	Catalytic site
3	Trp82	π-π stacking	CAS
3	Tyr332	π-π stacking	CAS
3	His438	Carbon Hydrogen bond	Catalytic site
3	Trp430	π- alkyl	Active-site gorge
3	Phe329	π-π stacking	CAS
3	Ala328	Alkyl	Active-site gorge
3	Tyr440	π- alkyl	Peripheral gorge
3	Gly116	Van der Waals	Peripheral gorge
3	Gly117	Van der Waals	Peripheral gorge
3	Pro285	Van der Waals	Peripheral gorge

Compound **3** adopts a well-packed orientation along the broad catalytic gorge of BChE, consistent with the favorable docking score (–10.2 kcal.mol⁻^1^). The pose is stabilized by a multimodal interaction network that combines aromatic stacking, hydrophobic contacts, and directional hydrogen bonds. Aromatic π–π/π–T interactions between the ligand’s fused rings and aromatic residues lining the anionic region of BChE (notably Gly 116 and Tyr332) provide primary anchoring and define the ligand’s trajectory toward the catalytic center. Additional π–alkyl/alkyl contacts with hydrophobic side chains within the acyl-binding pocket (e.g., Ala328, Trp82, Trp 430, and Tyr 440) contribute to tight packing and explain the low entropic penalty of the bound state. One or more conventional and carbon-hydrogen bonds with backbone atoms near the catalytic triad (Pro285) and the oxyanion site further orient the scaffold for optimal complementarity. Extensive van der Waals contacts along the gorge complete a continuous interaction surface from the peripheral entrance (Gly 116, Gly 117, and Pro285 region) toward the esteratic site, suggesting dual-region engagement within BChE (Fig. 7B, Table 4).

Taken together, these interactions rationalize the high predicted inhibitory potency and indicate that the compound is capable of simultaneously exploiting the anionic/peripheral zone and the esteratic subsite, a binding pattern frequently associated with potent BChE inhibitors. When considered alongside the AChE docking results (–10.5 to –11.3 kcal·mol⁻^1^ in this series), the present BChE score suggests comparable sub-micromolar binding propensity, with the broader BChE gorge likely favoring π-driven and hydrophobic stabilization over strictly catalytic-site anchoring. This profile is compatible with enzyme-tunable selectivity and can be leveraged in SAR to bias inhibition toward BChE by reinforcing distal aromatic contacts and maintaining at least one directional hydrogen bond near the catalytic region. To ensure the accuracy of docking study, validation was carried by re-docking the co-crystallized ligand (For hBChE, PDB ID: 5NN0). The predicted binding energy (ΔG) of the redocked ligand (N-((1-(2,3-dihydro-1H-inden-2-yl)piperidin-3-yl)methyl)-N-(2-(dimethylamino)ethyl)−2-naphthamide) was − 12.7 kcal/mol.

Despite the promising inhibitory and selectivity profiles observed for the investigated pyrimidine derivatives, several limitations of the present study should be acknowledged. First, the number of tested compounds was relatively limited (*n* = 7), which is typical for exploratory SAR investigations but may restrict statistical generalization of the observed activity trends. In addition, the molecular docking approach employed here provides static representations of ligand–enzyme interactions and therefore does not fully account for protein flexibility, solvent effects, or time-dependent conformational dynamics that may influence ligand binding. Consequently, docking results should be interpreted as qualitative structural hypotheses rather than definitive descriptions of ligand binding. Furthermore, the enzymatic inhibition experiments were performed using purified enzyme systems, which are appropriate for mechanistic characterization but do not reflect pharmacokinetic factors, metabolic stability, blood–brain barrier permeability, or potential off-target effects in complex biological environments. Finally, cholinesterases from different species exhibit documented sequence and structural variations that may influence ligand recognition despite overall conservation of the catalytic gorge. Therefore, further studies including expanded compound libraries, molecular dynamics simulations, and cell-based or in vivo evaluations will be necessary to comprehensively validate the therapeutic potential and binding mechanisms of these compounds.

## Conclusion

In conclusion, in this study, the inhibitory potential of pyrimidine derivative molecules **1–7** against AChE/BChE enzymes was evaluated using both in vitro inhibition and molecular modeling methods. Molecules **1–7** were determined to have high inhibitory potential and selectivity, particularly on AChE activity. Molecule **6** exhibited significant inhibitory potential for AChE, while molecule **3** exhibited significant inhibitory potential for BChE. Correlation and regression confirmed that molecules **1–7** exhibited a selective profile biased toward AChE, while molecular docking results indicated that molecules **1–7** exhibited favorable binding energies by forming multiple interactions with both the CAS and PAS regions of AChE. These results will contribute to studies on the development of new pyrimidine-based AChE/BChE inhibitors.

## Supplementary Information

Below is the link to the electronic supplementary material.

ESM 1 (DOCX 63.3 KB)

## Data Availability

All source data for this work (or generated in this study) are available upon reasonable request.
