# Supplementary material for: Evaluation of pyrimidine-based compounds as AChE and BChE inhibitors: in vitro inhibition, molecular modeling, and statistical evaluation
Source: Naunyn Schmiedebergs Arch Pharmacol. 2026 Apr 23;399(10):15227–39. doi: 10.1007/s00210-026-05347-0 (PMC13391709; doi:10.1007/s00210-026-05347-0)
Supplement: Supplementary file 1 — (DOCX 63.3 KB) [file 210_2026_5347_MOESM1_ESM.docx]

**Supplemantary File**

**Evaluation of Pyrimidine-Based Compounds as AChE and BChE Inhibitors: *In Vitro* Inhibition, Molecular Modeling, and Statistical Evaluation**

**Zuhal Alım^1^, Yeliz Demir^2,3*^**


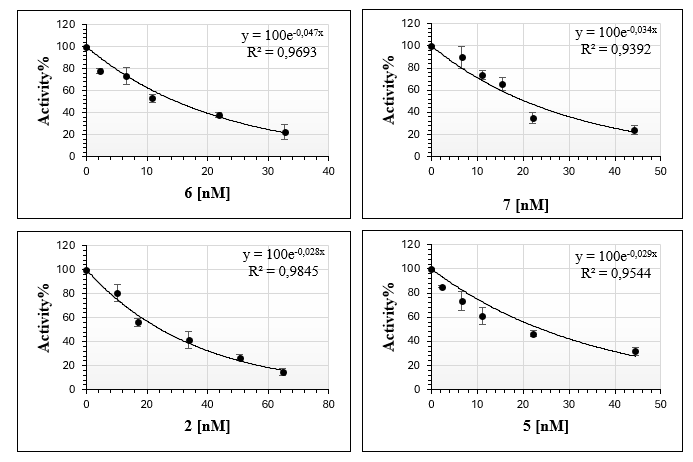


**Figure S1.** IC_50_ plots of the molecules that exhibited stronger inhibitory effects on AChE activity.


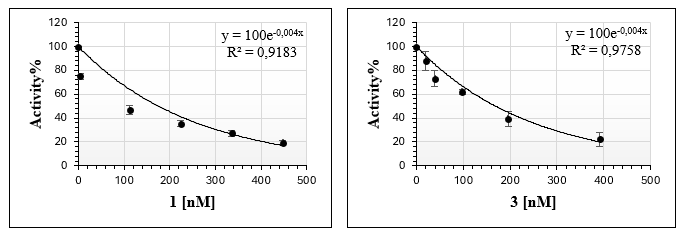


**Figure S2.** IC_50_ plots of the molecules exhibiting the strongest inhibition of BChE activity.
